# Probing Morbillivirus Antisera Neutralization Using Functional Chimerism between Measles Virus and Canine Distemper Virus Envelope Glycoproteins

**DOI:** 10.3390/v11080688

**Published:** 2019-07-27

**Authors:** Miguel Angel Muñoz-Alía, Stephen J. Russell

**Affiliations:** 1 Department of Molecular Medicine, Mayo Clinic, 200 First St SW, Rochester, MN 55905, USA; 2 Division of Hematology, Mayo Clinic, Rochester, MN 55905, USA

**Keywords:** chimeras, fusion protein, hemagglutinin protein, neutralizing antibodies, paramyxovirus, vaccines

## Abstract

Measles virus (MeV) is monotypic. Live virus challenge provokes a broadly protective humoral immune response that neutralizes all known measles genotypes. The two surface glycoproteins, H and F, mediate virus attachment and entry, respectively, and neutralizing antibodies to H are considered the main correlate of protection. Herein, we made improvements to the MeV reverse genetics system and generated a panel of recombinant MeVs in which the globular head domain or stalk region of the H glycoprotein or the entire F protein, or both, were substituted with the corresponding protein domains from canine distemper virus (CDV), a closely related morbillivirus that resists neutralization by measles-immune sera. The viruses were tested for sensitivity to human or guinea pig neutralizing anti-MeV antisera and to ferret anti-CDV antisera. Virus neutralization was mediated by antibodies to both H and F proteins, with H being immunodominant in the case of MeV and F being so in the case of CDV. Additionally, the globular head domains of both MeV and CDV H proteins were immunodominant over their stalk regions. These data shed further light on the factors constraining the evolution of new morbillivirus serotypes.

## 1. Introduction

Morbilliviruses, of the *Paramyxoviridae* family, are highly infectious respiratory viruses and cause devastating disease in humans and animals (recently reviewed by Pfeffermann et al. [1]). Measles virus (MeV) is the prototype morbillivirus and infects humans exclusively. In contrast, canine distemper virus (CDV) is a rather promiscuous carnivore morbillivirus, able to infect even primates and cause a disease outbreak [2,3]. This characteristic poses a more than theoretical risk that CDV could extend into the human population after MeV eradication [4,5,6]. Even though high titers of MeV-neutralizing antibodies have been shown to protect against CDV infection [7], those levels are not routinely achieved through vaccination [4].

The morbillivirus envelope comprises two envelope glycoproteins that encompass the so-called virus fusion-membrane apparatus: the attachment (H) and the fusion (F). The attachment glycoprotein is required for the initial binding step to the host cell receptor, whereas the F glycoprotein mediates the fusion event between the viral and host cell membranes. F glycoprotein also can fuse adjacent cell membranes, forming syncytia when cells expressing H and F interact with cells expressing the receptors [8,9]. The signaling lymphocytic activation molecule family member 1 (SLAMF1) on immune cells and the nectin cell adhesion molecule 4 (NECTIN-4) in epithelial cells are the known receptors in morbilliviruses [10,11]. Vaccine strains of MeV and CDV have gained the ability to infect other cell types in a CD46-dependent and independent manner, respectively [12,13,14,15,16]. Cross-talk between H and F seems virus-species specific, and albeit heterologous combinations typically compromise fusion activity, successful examples exist [17,18,19,20,21].

Targeting virus surface proteins and neutralization antibodies are crucial for vaccine-bestowed protection [22]. On the basis of epidemiologic studies, a microneutralization titer of 210 mIU/mL or greater has been assigned as an absolute correlate of protection against measles [23,24,25]. The knowledge on the antigenic determinants recognized after natural infection or vaccination has depended heavily on the availability of monoclonal antibodies and DNA-based vaccines [18,26,27,28,29,30,31,32]. Since the morbillivirus coat consists of hetero-oligomers of the H and F glycoproteins that are organized into tetramers and trimers, respectively, single expression of antigens could alter the overall immunogenicity of the protein [33,34]. Moreover, the hybridoma technology is known to be subjected to bias, and therefore their representativeness with the in vivo immune responses is questioned [35,36]. Thus, the exact antigenic determinants involved in morbillivirus protection are poorly characterized.

To address this gap in the knowledge, we used an improved reverse genetic system to generate recombinant MeV-encoding chimeric envelope glycoprotein from the close, but not cross-reactive, CDV. With the use of virus-specific antisera induced in different species, our study provides the neutralizing-antibody response against the virus envelope glycoproteins.

## 2. Materials and Methods

### 2.1. Cell Lines

Baby hamster kidney cells (BHK; american type culture collection [ATCC], Cat. #CCL-10, Manassas, VA, USA) and Vero African green monkey kidney cells (Vero; ATCC, Cat. #CCL-81) and their derivatives (either expressing human nectin-4 [37], canine SLAM [38], or a membrane-anchored single-chain antibody specific for a hexahistidine peptide [39]) were kept in Dulbecco’s modified Eagle’s medium (DMEM) (GE Healthcare Life Sciences, Cat. #SH30022.01, Pittsburg, PA, United States). The human melanoma Mel-JuSo cell line was a kind gift from Rik de Swart, PhD (Erasmus MC, Rotterdam, The Netherlands), and was grown in Roswell Park Memorial Institute (RPMI) 1640 medium (Corning Inc, Cat. #10-040-CV, Corning, NY, United States). Cells were supplemented with 1 × penicillin/streptomycin (Corning Inc, Cat. #30-002-CI) and 5 mM HEPES (Life Technologies, Cat. #15630-080, Carlsbad, CA, United States). Additionally, Vero, Vero/anti-His, and Vero/NECTIN-4 were maintained in the presence of 5% fetal bovine serum (FBS) (Thermo Fisher Scientific, Cat. #10437-028, Waltham, MA, United States). BHK, Mel-JuSo, and Vero/dogSLAM cells were grown in 10% FBS plus 1 mg/mL of Zeocin (InvivoGen, San Diego, CA, United States) (Vero/dogSLAM). Vero/NECTIN-4 cells were propagated in the presence of 0.5 mg/mL G418 (Mediatech Inc, Cat. #MT-61-234-RG, Manassas, VA, United States). Cells were kept at 37 °C and 5% CO_2_ with saturating humidity.

### 2.2. Construction of Plasmids

The chimeric MeV/CDV-H (MeV-H stalk combined with CDV-H head domain) and CDV/MeV-H (CDV-H stalk combined with MeV-H head domain) genes were generated through overextension polymerase chain reaction (PCR). For the MeV/CDV-H chimera, the amino acid (aa) 1–159 portion of vaccine-lineage MeV-H Nse strain [40,41] was amplified with phusion high-fidelity DNA polymerase (Thermo Fisher Scientific, Cat. #F-530S) with primers V181 (+) [42] and 5′-CTG TGC AGA TGT GGC TGC TAG AAA AGC TAT TGC ATC GGC AGC A-3′. The aa 160-604 portion of CDV-H (strain Onderstepoort) [20] was amplified with the pair of primers 5′-CTG TGC AGA TGT GGC TGC TAG AAA AGC TAT TGC ATC GGC AGCA-3′ and pCG-H(−): 5′-CCA GAA GTC AGA TGC TCA AGG G-3′. For the CDV-H/MeV-H chimera, the CDV-H portion was amplified with V181(+) and 5′-TCA CCA ATG CAT TCA TGA GCT CTT CGA TCC CAA TTG ACT CAC AGT AAT TAG-3′, whereas the pair 5′-ACT AAT TAC TGT GAG TCA ATT GGG ATC GAA GAG CTC ATG AAT GCA TTG GTG A-3′ and pCG-H(−) was used for the MeV-H portion. Finally, PCR products were hybridized and amplified with the pair of primers V181 (+)/pCG-H(−) and cloned into pJET1.2/blunt vector (Thermo Fisher Scientific, Cat. #K1241). 

Site-directed mutagenesis (QuikChange Site-Directed Mutagenesis Kit; Agilent Technologies, Santa Clara, CA, United States) was used to remove a SpeI site in CDV-H and to introduce a Y537D substitution, shown to diminish neutralization activity in human serum [7]. All constructs were C-terminally fused to 6 × His and subcloned into pCG-H plasmid via PacI/SpeI sites [40]. Alternatively, the GCN4(7P14P) peptide of the sequence AHLENEVARLK was inserted instead of the 6 × His tag. 

To generate the pseudoreceptor for the GCN4(7P14P) peptide, the gene encoding the single-chain variable fragment (scFv, clone C11L34) [43] was synthesized and cloned with In-Fusion HD Cloning Kit (Takara Bio Inc) into the SfiI/AccI site of the pDisplay vector (Thermo Fisher Scientific, Cat. #V660-20). The same sites were used previously to clone the anti-His tag scFv in frame with the immunoglobulin (Ig) κ-chain leader sequence, hemagglutinin (HA) epitope, and platelet-derived growth factor receptor transmembrane domain found in the vector [39].

### 2.3. Fusion Assay

For quantitative fusion assay, Expi293F cells (Thermo Fisher Scientific, Cat. #A14527) were used as effector cells and cotransfected with 150 ng each of H and F and the second part of the dual-split reporter plasmid (DSP_8-11_) [44,45] with use of ExpiFectamine293 (Thermo Fisher Scientific, Cat. #A14524). One day later, separate 6-well plates containing Vero cells (target cells) and their derivatives expressing the different receptors were transfected with 1 μg of DSP_1-7_ with use of TransIT-LT1 transfection reagent (Mirus Bio LLC). Then, 1 day later, Vero cells were detached and mixed with Expi293F cells (1:2 ratio) in DMEM/F-12 (Thermo Fisher Scientific, Cat. #11330-021) containing 60 μM EnduRen Live Cell Substrate (Promega Corp, Cat. #6481, Madison, WI, United States). In each well of a black 96-well plate (Corning Inc, Cat. #3603), 4.8 × 10^5^ cells were distributed and incubated at 37 °C with 5% CO_2_. Content mixing was monitored over 12 h with an Infinite M200 Pro multimode microplate reader (Tecan Trading AG). The experiment was repeated twice with 3 intraexperiment replicates.

For the determination of binding affinity in fusion activity, BHK cells were used as effector cells and overlaid with Vero cells that were additionally transfected with 2 μg of scFv 6 × His or GCN4 expression plasmid.

For visual assessment of fusion, Vero cells and derivatives were transfected with 1 μg each of H and F proteins and stained 1 day later with Hema-Quik (Thermo Fisher Scientific, Cat. #123-745). Images were obtained with a microscope (Eclipse Ti-S; Nikon) at 4 × magnification.

### 2.4. Flow Cytometric Analysis

For assessment of the level of H protein expressed at the cell membrane level, cells were transfected and analyzed with flow cytometry as previously described [46]. Alternatively, Mel-JuSo cells were transfected with 2.5 μg of the appropriate construct. At one day posttransfection, cells were trypsinized and resuspended with fluorescent activated cell sorting (FACS) buffer (i.e., phosphate-buffered saline supplemented with 2% FBS) containing a 6 × His tag monoclonal antibody (Thermo Fisher Scientific, Cat. #MA1-135). After 30 min at 4 °C, cells were washed 3 times with FACS buffer and subsequently incubated with Alexa Fluor 594 goat anti-mouse IgG (H+L) cross-absorbed secondary antibody (Thermo Fisher Scientific, Cat. #A-11005) for another 30 min at 4 °C. This step was followed by another 3 washes and fixation with paraformaldehyde 1% (Electron Microscopy Sciences, Hatfield, PA, United States). The geometric mean fluorescence intensity was collected from 10,000 events with use of a flow cytometer (FACSCanto; BD Biosciences, San Jose, CA, United States). 

Antibodies were used to analyze receptor expression on the Vero cells derivatives, anti–nectin-4 (R&D Systems, Cat. #FAB2659P, Minneapolis, MN, United States) (Vero/NECTIN-4) or anti-HA (Thermo Fisher Scientific, Cat. #26183) (Vero/anti-His and Vero/dogSLAMF1). Data were analyzed with use of Cytobank Inc [47]. 

Quantification of the rescue efficiency was determined at 72 h post-transfection, following procedures reported previously [48].

### 2.5. Western Blot

Mel-JuSO cells were transfected with 2.5 μg of the appropriate plasmid and lysed 24 h later with 100 μL of 1 × radioimmunoprecipitation assay buffer (Abcam, Cat. #ab156034, Cambridge, United Kingdom). Reduced cell lysates were treated, or not, with PNGase F in accordance with the manufacturer’s instruction (New England Biolabs, Cat. #P0705S, Ipswich, MA, United States) and underwent Western analysis, as described previously [46], with mouse monoclonal antibody anti–6 × His tag (Thermo Fisher Scientific, Cat. #MA1-135), mouse monoclonal anti–MeV-H globular head (BH195) [49], rabbit polyclonal anti-MeV-H cytoplasmic tail [40], mouse anti–β-actin horseradish peroxidase (HRP)-conjugated (Sigma-Aldrich Co, Cat. #A3854), goat anti-mouse IgG (H+L) HRP-conjugated (Thermo Fisher Scientific, Cat. #62-6520), and goat anti-rabbit IgG (H+L) cross-absorbed HRP-conjugated (Thermo Fisher Scientific, Cat. #31462). Polyvinylidene fluoride (PVDF) membranes (Thermo Fisher Scientific, Cat. #IB24002) were reprobed after washing the PVDF membrane with stripping solution [50]. Membranes were scanned with a ChemiDoc Gel Imaging System (Bio-Rad Laboratories, Inc, Hercules, CA, United States) and analyzed with Image Lab Software (v 6.0.0 build 25; Bio-Rad Laboratories, Inc).

### 2.6. Recombinant Measles Viruses (rMeVs)

The recombinant vaccine lineage MeV-vac2 green fluorescent protein (GFP)N [51] was used as backbone for the generation of recombinant viruses. This vector expresses the enhanced GFP (EGFP) upstream of the N gene. To avoid plasmid instability during bacterial propagation, the plasmid backbone was replaced with the pSMART LC kan vector (Lucigen Corp, Cat. #40821-1, Middleton, WI, United States) in a stepwise manner after 2 approaches. In the first approach, a multicloning site with SacII and NotI restriction enzymes were added to the vector. Then, an optimal T7 promoter (T7_opt_) [52], followed by a hammerhead ribozyme (HHRbz) [48,52,53,54] [53,54], was inserted upstream of the viral genome with insertion of the sequence directly into the forward primer, amplifying the MeV genome to a unique internal restriction site SacII located at the beginning of the P gene. The SacII-NotI fragment in MeV-vac2(GFP)N vector was then inserted into the similarly digested pSMART LC kan vector. In the second approach, a cassette containing the human elongation factor was followed by a chimeric intron (pCI vector; Promega Corp, Cat. #E1731), T7_opt_, HHRbz, and cloning sites were synthesized and ligated into the vector. A SV40 polyadenylation signal was additionally inserted downstream of the viral antigenome. All plasmid propagations were performed in *Escherichia coli* Stbl2 cells (Thermo Fisher Scientific, Cat. #10268019) grown at 30 °C.

In the production of envelope-exchange MeVs, MeV-H was easily replaced with the specified H construct using PacI/SpeI sites in both vectors. To replace MeV-F from MeV antigenome plasmid, pCG-CDV-F [20] was digested with HpaI/SpeI and inserted into the similarly digested pCG-MeV-F [41]. The NarI/SpeI fragment of this plasmid was then used to replace that in the MeV-based vector.

The recovery of recombinant MeV (rMeV) was performed with cotransfection of rMeV antigenomic plasmid construct; N, P, and L supporting plasmids derived from a MeV isolate genotype B3.1 [55]; and a codon-optimized T7 RNA polymerase (a gift from Benhur Lee, Addgene; plasmid #65974), with Lipofectamine LTX transfection reagent (Thermo Fisher Scientific, Cat. #15338-100), as described previously [48]. Transfected cells were cocultured after 4 days with Vero/NECTIN-4 cells, and the virus was amplified thereafter through further infections at a multiplicity of infection 0.03. Virus titer was determined with use of fluorescence-forming units (FFUs). The identity of the recombinant MeVs was confirmed with Sanger sequencing after RNA extraction from infected cells as indicated previously [42]. 

### 2.7. Virus Growth Kinetic Analysis

Vero/NECTIN-4 cells were seeded in 6-well plates at 4 × 10^5^ cells per well at 16 to 18 h before infection. Cells were infected at a multiplicity of infection 0.03 in 1 mL of Opti-MEM (Thermo Fisher Scientific, Cat. #31985070). After a 90-min absorption period at 37 °C, virus inoculum was discarded and cells were washed 3 times with Dulbecco’s phosphate buffered saline (DPBS) (Mediatech, Inc, Cat. #MT-21-031-CVRF). Cells were replenished with fresh DMEM 5% FBS and scraped into the same well containing medium at the indicated time points, followed by 3 freeze–thaw cycles. Virus titers were determined with Vero/NECTIN-4 seeded at 10^4^ cells per well of a 96-well plate and read at 42 h postinfection.

### 2.8. Neutralization Assays

A plaque reduction microneutralization (PRMN) assay was carried out as described previously [42]. Briefly, Vero/NECTIN-4 and Vero/dogSLAMF1 cells were seeded the day before the experiment at 10^4^ cells per well of a 96-well plate. Serial 2-fold dilution of the corresponding heat-inactivated antisera (56 °C for 30 min) was mixed with an equal volume of 90-FFU virus (calculated in the corresponding cell line to be used) in Opti-MEM. The serum–virus mixture (55 μL, 4 replicates per dilution) was incubated for 1 h at 37 °C and added to recently confluent cells. After an absorption period of 90 min, cells were supplemented with DMEM 5% FBS, and infection was left to proceed for 40 h at 37 °C and 5% CO_2_.

Control wells (i.e., virus without serum) were averaged to represent 100% relative infection. The neutralization dose 50 was calculated with a sigmoidal dose response curve (Prism 7.0; GraphPad Software, Inc). When the data could not be fitted (“not converge”), curves were not drawn. Inclusion of the World Health Organization’s Third International Standard for antimeasles antibody enabled the conversion of antibody titers to mIU/mL through direct comparison with the antibody titers [24]. 

Human sera used in the present study was pooled from 60 to 80 donors who are specifically blood type AB (Valley Biomedical Products and Services, Inc, Cat. #HS1017, Lot #C80553). The following reagents were obtained through the National Institutes of Health (NIH), Biodefense and Emerging Infections Research Resources Repository, and the National Institute of Allergy and Infectious Diseases, NIH: 1) polyclonal anti-MeV, Edmonston (antiserum, guinea pig), NR-4024. This antisera was produced via two hydropulses with the Edmonston strain grown on BS-C-1 cells in 0.5% lactal and 10% FBS. 2) polyclonal anti–CDV, Lederle avirulent (antiserum, ferret), NR-4025. This antisera was produced adding 10% sucrose gelatin to the virus grown in chicken embryo fibroblast. On the basis of the amount of antiserum available at the repository under the same lot number, they likely correspond to a pool of antiserum samples.

### 2.9. Statistical Analysis

Statistical analysis was performed with statistical software (Prism version 7.0a; GraphPad Software, Inc, San Diego, CA, United States). Statistical significance between groups was determined with use of 1-way analysis of variance with Bonferroni’s multiple comparison test.

## 3. Results

### 3.1. Expression and Characterization of Hybrid H Constructs

**Figure 1:**
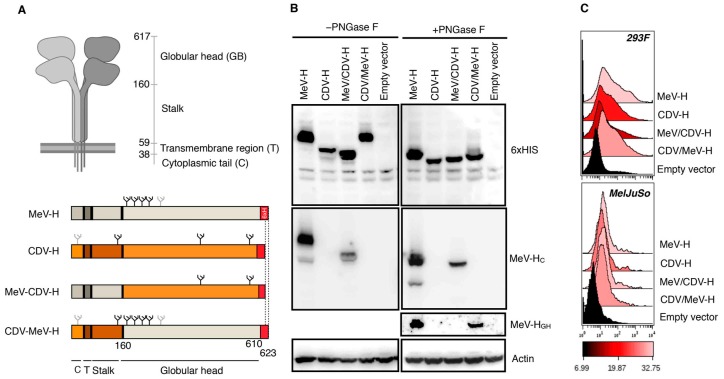
Folding and surface expression of hybrid H constructs. (**A**) Diagram of measles virus H glycoproteins. Created with BioRender.com. H hybrid proteins were constructed by swapping the globular head of the MeV-H or CDV-H among each other, using residue 160 as the delineating point. All hybrids were additionally tagged with a hexahistidine peptide (6 × HIS). Glycans present in the H constructs are indicated and are depicted as black if known to be used, or as gray otherwise. (**B**) Western blot analysis of hybrid constructs. MelJu-So cells were transfected with the appropriate plasmid, and cell lysates were deglycosylated with PNGase F. Samples were then analyzed by sodium dodecyl sulphate-polyacrylamide gel electrophoresis and immunoblotted with the indicated antibodies. (**C**) FACS analysis results for cell surface expression. H expression on transiently transfected Expi293F or MelJu-So cells was analyzed through flow cytometry with anti-6 × His antibody. Color coding indicates geometric mean. CDV indicates canine distemper virus; CDV-H, canine distemper virus–H glycoprotein; FACS, fluorescent-activated cell sorting; 6 × H, 6 × HIS; MeV, measles virus; MeV-H, measles virus–H glycoprotein; MeV-H_C_, measles virus–H glycoprotein anti–cytoplasmic tail; MeV-H_GH_, measles virus–H glycoprotein anti–globular head. (Used with permission of Mayo Foundation for Medical Education and Research).

Hybrids of H proteins are expected to stay functional, on the basis of our experience in heterotypic fusion activity when both vaccine strains of MeV (Edmonston) and CDV (Onderstepoort) were used [42]. The H glycoprotein is a type II integral homotetrameric protein consisting of a large ectodomain structured as a helical stalk and a 6-blade, β-propeller, folded globular head that harbors the receptor-binding site. By defining residue 160 as the delineating point between the head and the stalk domain, we constructed two hybrid H proteins encompassing the amino-terminal cytoplasmic tail, transmembrane domain, and stalk region with the corresponding heterologous head domain from either MeV-H or CDV-H (named hereafter CDV/MeV-H and MeV/CDV-H, respectively) (Figure 1A). After inserting a histidine tag at the C-termini, we tested with Western blot and flow cytometry whether the different chimera were properly folded and expressed on the cell surface, respectively (Figure 1B,C). 

Of note, MeV-H globular head is enlarged by 13 aa compared with the homolog CDV-H. Furthermore, MeV-H contains four N-linked glycosylation sites (NGS) (N168, N187, N200, and N215), whereas CDV-H contains three NGS (N149, N422, and N587). Accordingly, MeV-H and CDV-H could easily be distinguished by the lower apparent molecular weight of the latter (Figure 1B). MeV/CDV-H showed a slightly faster migration pattern than the parental CDV-H, likely because of the loss of the N149-linked glycosylation in the chimera. Furthermore, we confirm chimerism successfully by probing with an anti–MeV-H cytoplasmic tail antibody. The CDV/MeV-H chimera, which conserved all four NGS from the parental MeV-H, showed similar migration on the gel. Expression of the MeV-H globular head atop a CDV-H stalk was further confirmed with an anti–MeV-H antibody targeting the globular head. As expected, the previous anti–MeV-H cytoplasmic tail antibody failed to recognize such chimeric H. All constructs showed a similar migration pattern when treated with PNGase, indicating that differences in migration pattern were largely because of N-linked glycosylation. 

To examine whether the different constructs could traffic to the cell surface similar to the parental constructs, we performed flow cytometric analysis on two different transiently transfected cells (Figure 1C). With this method, all constructs could be detected on the cell surface, with comparable levels across the cell lines. 

### 3.2. Functional and Heterotypic Interaction of Hybrid H Proteins

**Figure 2:**
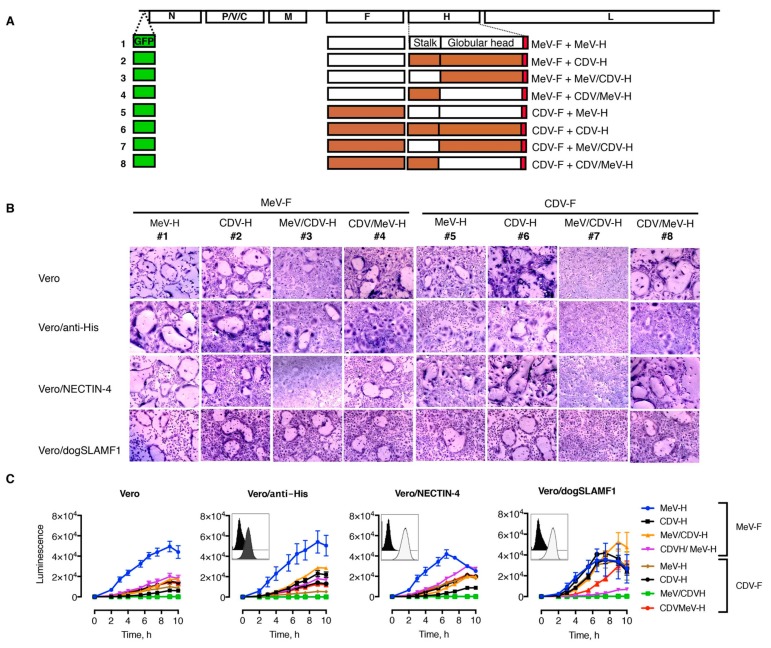
Functional analysis of hybrids construct and heterotypic complementation. (**A**) Schematic representation of the morbillivirus genome organization. Open boxes represent the open reading frames: nucleocapsid (N), phospho- (P), matrix (M), fusion (F), hemagglutinin (H), and large (L). V and C are accessory proteins transcribed from the P gene. Combinations of H and F proteins were cotransfected for functional assays and incorporated afterward into the MeV genome. Combinations and virus numbers denote the combination of the envelope glycoproteins used. (**B**) Syncytium formation assay. These representative microphotographs were taken at 24 h after transfection of cells with plasmid DNA encoding various H and F protein combinations. Scale bar represents 100 μm. (**C**) Quantitative fusion assay. Expi293F cells (effector cells) were transfected with the different plasmids encoding H and F plus one part of the dual-split reporter plasmid (DSP). After cocultivation with Vero cells carrying the second DSP half, the luminescence signal was recorded over time. Values and error bars (SDs) are from one representative experiment performed in triplicate. Insert indicates surface expression of the corresponding Vero cell derivate. CDV indicates canine distemper virus; CDV-F, canine distemper virus–F glycoprotein; CDV-H, canine distemper virus–H glycoprotein; MeV, measles virus; MeV-F, measles virus–F glycoprotein; MeV-H, measles virus–H glycoprotein. (Used with permission of Mayo Foundation for Medical Education and Research).

Having seen that H constructs can be expressed and transported to the cell surface, we next wanted to assess whether hybrid constructs stayed fusion competent. To test this, cell–cell fusion formation was first qualitatively analyzed after transfection with different combinations of H and F expression plasmids (Figure 2A). Given the interplay between receptor-binding-affinity and intercellular fusion [56], we used cultures of Vero (CD46+), Vero/anti-His (CD46+, anti-His scFv), Vero/NECTIN-4 (CD46+, NECTIN-4+), and Vero/dogSLAMF1 (CD46+, SLAMF1+) cells. The histidine tag displayed at the C-terminus domain of H can replace the requirement of natural receptor on Vero cells [39] and was chosen as an equalizer for fusion proficiency of the constructs in a receptor-dependent manner.

With regard to the morbillivirus natural receptors, human vs canine nectin-4 was preferred since CDV-induced fusion has been shown to be highly efficient [37]. Similarly, canine SLAMF1 can be used for MeV, whereas CDV-induced fusion is severely restricted when paired with the human SLAMF1 noncognate receptor [3,18]. In Figure 2B, we illustrate heterotypic complementation between combinations of MeV and CDV H and F proteins. The chimeric CDV/MeV-H construct was able to trigger MeV-F and CDV-F mediated fusion (combinations 4 and 8, respectively) in the different cell lines, although different efficiencies were noticed. Yet, cell–cell fusion induced by the chimeric MeV/CDV-H construct (combinations 3 and 7) was substantially decreased. The effect was more profound when paired with CDV-F (combination 7). 

To assess the fusion properties more specifically, we next used a quantitative cell–cell fusion assay based on a self-associating split luciferase assay [44,45]. In this case, on content mixing between effector cells expressing fusion machinery (H and F) and target cells expressing the receptors, the otherwise nonfunctional halves of the luciferase self-associate and reconstitute the enzyme activity, which can be measured in real time through permeable substrates. Because CDV-H induced fusion in all cell lines that we tested in a yet-unidentified receptor, we used suspension cells as effector cells (CD46+). The gentle swirl of the cells to keep them in suspension efficiently hindered early fusion events, then allowing for efficient detection mixing content when cocultured with the target cells (Figure 2C). Despite the fact that the homotypic CDV-H/F combination (combination 6) is known to induce fusion in Vero cells in a CD46-independent manner, the fusion activity was increased with cells expressing NECTIN-4 and SLAMF1, thereby indicating the preference of these receptors. On the contrary, no differences were observed on whether Vero cells expressed the pseudoreceptor for the 6 × His peptide on the construct. The homotypic MeV-H/F combination (combination 1) yielded higher luciferase levels than the homotypic CDV-H/F combination. However, their fusogenicity was comparable in SLAMF1-expressing cells. A general trend toward increased fusion activity was observed for the hybrid H proteins when either NECTIN-4 or SLAMF1 was present. This was particularly noticeable for the combination of MeV/CDV-H with MeV-F (combination 3). However, the combination of CDV/MeV-H protein with MeV-F (combination 4) led to increased fusion levels on NECTIN-4, and the pair MeV/CDV-H and CDV-F (combination 7) did not induce tangible fusion activity in any cell line. 

### 3.3. Increased Binding Affinity for a Pseudeoreceptor Does Not Improve Fusion Activity

Since receptor affinity is considered a major determinant of fusion activity, we next assessed whether an increase in the binding affinity of the peptide–antipeptide scFv system would result in fusion activity levels comparable to those observed when NECTIN-4 and SLAMF1 were present. To this aim, we substituted the 6 × His peptide for AHLENEVARLK, which is derived from the yeast transcription factor GCN4 [43]. This peptide was chosen because of the availability of the crystallographic structure of scFv binders with picomolar affinity [43]. Since the interaction of the 6 × His peptide to the scFv (Kd) is in the nM range [57], detection of differences in cell–cell fusion promotion would be the consequence of the receptor affinity. 

**Figure 3:**
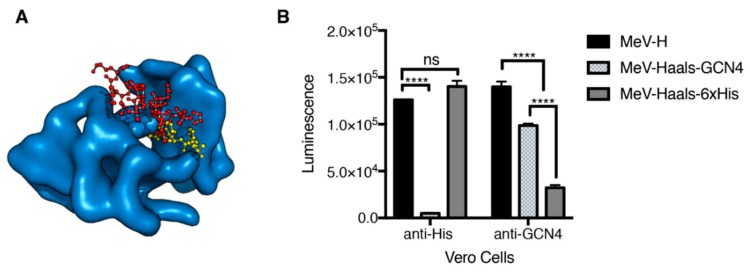
Effect of affinity binding in pseudoreceptor fusion levels. (**A**) Crystallographic structures for the tested peptide–pseudoreceptor systems. Crystallographic structures for the scFv binder to GCN4(7P-14P) peptide (PDB [Protein Data Bank] ID 1p4b) with the position of the His-peptide binding are shown (based on structural overlay with the scFv-His peptide structure, PDB ID 1kTR). The scFv binder is shown in a blue surface representation. GCN4(7P-14P) and His peptide binders are shown as red and yellow balls and spheres, respectively. (**B**) A quantitative fusion assay was performed with baby hamster kidney cells bearing the indicated H protein with MeV-F. Vero cells that were transfected with the corresponding pseudoreceptor were used as effector cells. Haals indicates an H protein blind for the natural receptors. Maximum values over a 10-h experiment are shown, with mean (SD) of a representative experiment performed in triplicate. **** indicates *p* <.0001. MeV-F indicates measles virus–F glycoprotein; MeV-H, measles virus–H glycoprotein; ns, no significance, determined through 1-way analysis of variance with Bonferroni’s multiple comparison test (Used with permission of Mayo Foundation for Medical Education and Research).

For this experiment, we used BHK cells coexpressing MeV-F with MeV-H 6 × His or GCN4 tagged, which were overlaid with Vero cells expressing scFv 6 × His or GCN4. To ascertain that cell–cell fusion was the consequence of peptide–pseudoreceptor interaction, we introduced, into the tagged MeV-H, protein mutations that ablate natural tropism (MeV-Haals; Y481A, R533A, S548L, F549S) [39] and compared them with that obtained by the untagged MeV-H on binding to CD46. When Vero cells expressed the 6 × His pseudoreceptor, fusion was promoted for MeV-H and MeV-Haals-6 × His but not MeV-Haals-GCN4, confirming detargeting and concomitant retargeting of MeV-H (Figure 3). Since MeV-H activity in Vero cells is mediated by CD46 binding, the His-pseudoreceptor system provides sufficient energy to activate fusion. For Vero cells expressing scFv GCN4 pseudoreceptor, MeV-H-6 × His did not induce fusion, but MeV-Haals GCN4 did. However, MeV-Haals GCN4 achieved significantly lower fusion levels than MeV-H or MeV-Haals-6 × His in receptor-positive cells. Therefore, an increase in binding affinity in the context of a pseudoreceptor system does not increase fusion levels. Hence, other factors must contribute to the enhanced cell–cell fusion activity observed in a NECTIN-4 and SLAMF1-dependent manner.

### 3.4. Generation of rMeV Bearing Different Hybrid H Proteins and F Combinations

**Figure 4:**
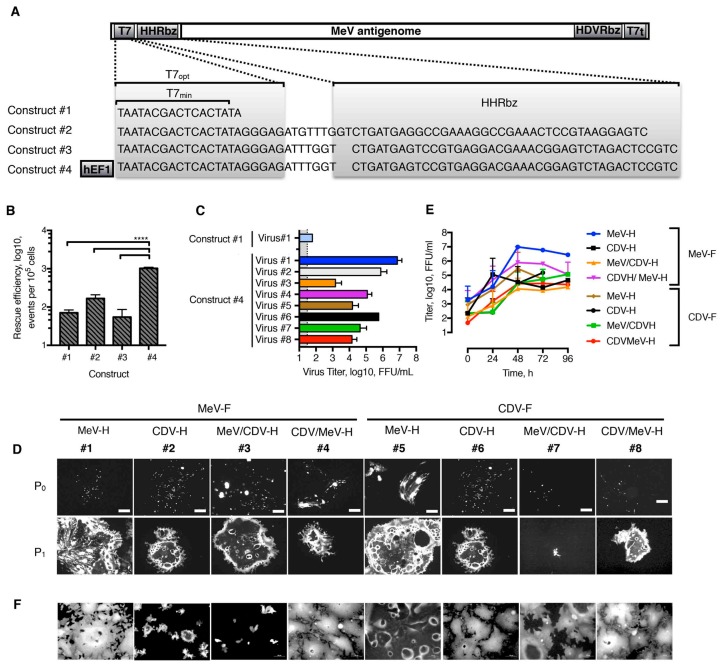
Rescue and in vitro characterization of chimeric viruses. (**A**) Sequence of the T7 promoter, variable sequence, and HHRbz tested in the context of the plasmid carrying the MeV antigenome. T7_min_ is the minimal T7 promoter without additional 3Gs. T7_opt_ is an optimal T7 promoter that has an additional AGA sequence to the 3Gs. This T7_opt_ has been shown to enhance transcriptional levels [52]. HHRbz in construct #1 [53], as well as those in constructs #2 and #3 [48], has been used as described. (**B**) Rescue efficiency (i.e., number of successful rescue events per transfected cells). This efficiency was determined through flow cytometric analysis of rescue cells at 3 days posttransfection to determine the number of EGFP-positive cells. Results from three independent experiments are shown. **** indicates *p* <.0001, as determined in 1-way analysis of variance. (**C**), Virus production at P_1_. Rescue cells were overlaid at 4 days posttransfection onto Vero/NECTIN-4 and incubated for another 3 days at 37 °C with 5% CO_2_. Cells were subjected to three freeze–thaw cycles and the supernatant was titered onto Vero/NECTIN-4 cells. Construct #4 was used as a backbone for rMeVs. MeV in construct #1 was included for comparison of virus production. (**D)** Rescue monitoring of rMeVs. EGFP autofluorescence was determined on rescue cells at 4 days after transfection (P_0_). Next, they were overlaid onto Vero/NECTIN-4 and further incubated for 3 days (P_1_). (**E)** Growth curve analysis of rMeVs. Vero/NECTIN-4 cells were infected at an MOI 0.03, and whole-well content was harvested at the indicated time points. Values and error bars represent SD from 2 independent experiments. (F) EGFP autofluorescence of Vero/NECTIN-4 cells at 48 h postinfection and at MOI 0.03 with the indicated rMeV. Scale bar represents 100 μm. EGFP indicates enhanced green fluorescent protein; FFU, fluorescence forming unit; HDVRbz, hepatitis delta virus ribozyme; hEF1, human elongation factor-1α core promoter; HHRbz, hammerhead ribozyme; MeV, measles virus; MOI, multiplicity of infection; rMeV, recombinant measles virus; T7t, T7 terminator. (Used with permission of Mayo Foundation for Medical Education and Research).

We had determined that hybrid H proteins are properly folded and can transmit the fusion-triggering signal to heterologous F proteins. Therefore, we next pursued the rescue of such chimeric viruses (Figure 2A). Since the *Paramyxoviridae* reverse genetic system has low rescue efficiency [48], we first looked to improve the robustness and efficiency of the MeV rescue system on the basis of genetic modification of the MeV-containing plasmid. To start, the minimal T7 promoter found in the original plasmid was replaced with an optimal T7 promoter, as described by Yun et al. [52]. To ensure compliance with the Paramyxoviruses’ “rule of 6” (the number of nucleotides in the genome must be of polyhexameric length to allow efficient replication), we also introduced self-cleaving HHRbz. We screened for two different HHRbz, previously shown to be functional [52,53]. In addition, we combined the use of T7_opt_ with a human Pol II promoter. We therefore built four different versions containing the MeV antigenome flanked at 3′ with the hepatitis delta virus ribozyme (HDVRbz) and T7 terminator (T7t) (Figure 4A). The effectiveness of the approach was tested through analysis of GFP-positiveness of single-step transfected cells. Whereas HHRbz swap did not significantly affect rescue efficiency, the further addition of a human Pol II promoter increased it by approximately 1 log, which was statistically significant (Figure 4B). Interestingly, virus production was improved by 6-log or more with the use of construct #4 over construct #1 (Figure 4C, virus #1). On the basis of our previous results and the greater conservation of nectin-4 over SLAMF1 (94% and 65%, respectively) [37,58] between humans and dogs, the rescue of the different rMeV was attempted on Vero/NECTIN-4 (Figure 4D). Thus, it was not expected that rMeV expressing a MeV-H globular head would adapt to use more efficiently than an otherwise noncognate SLAMF1, a fact that would influence neutralization sensitivity [58]. We succeeded in rescuing all chimeric MeV, although with up to 4-log differences in viral titers at P_1_. The identity of the rMeV was then confirmed by reverse transcriptase–PCR and Sanger sequencing. These methods showed the expected identity for H and F glycoproteins. Next, viruses were further propagated on Vero/NECTIN-4 cells and the growth characteristics were analyzed. Unlike the parental MeV, all chimeric rMeV grew at lower titers (Figure 4E). The isogenic MeV expressing CDV envelope glycoproteins peaked at 24 hpi, decreasing its viral titers afterwards due to massive syncytia formation. The rest of rMeV showed peak titers at 48 h post-infection, with the further exception of construct #7, which expressed the hybrids MeV/CDV-H proteins with CDV-F. All in all, rMeV can display functional chimeric envelope glycoproteins.

### 3.5. Neutralization Profile of Virus-Specific Antisera in Different Species

Finally, we wanted to test whether the different isogenic rMeVs were able to identify antigenic determinants on the morbillivirus coat. To answer this question, we used measles-immune antisera from guinea pigs and humans. For CDV-immune antisera, we used the ferret model, highly applied in pathogenesis studies [38]. With these antisera, we performed PRMN assay with the different isogenic rMeVs on NECTIN-4 and SLAMF1-expressing cells. Of note, neutralization titers were highest when probed on NECTIN-4 cells. However, no differences were noted on the neutralization profiles. 

**Figure 5:**
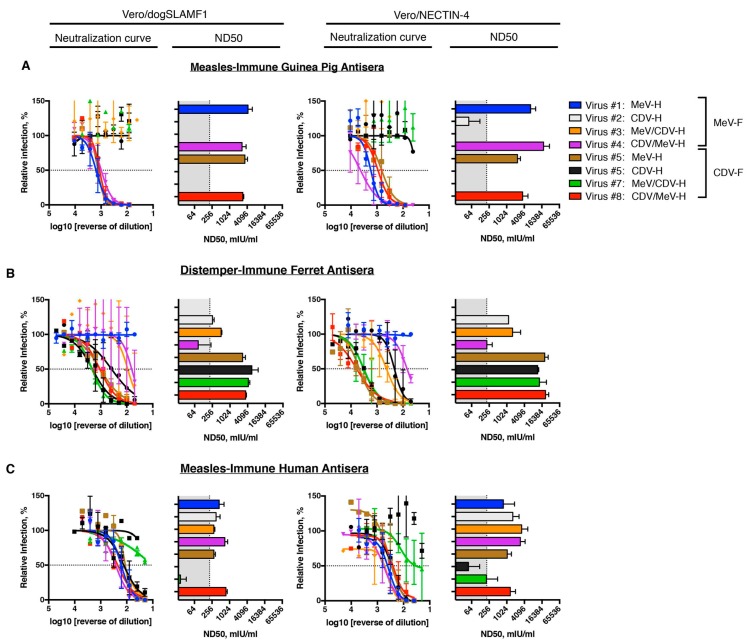
Species–specific plaque reduction microneutralization (PRMN) profiling. (**A**) PRMN activity of measles virus-immune (guinea pig) antisera. (**B**) PRMN activity of canine distemper virus-immune (ferret) antisera. (**C**) PRMN activity of measles virus-immune (human) antisera. PRMN was performed on the indicated cell lines. Briefly, a fixed amount of virus was preincubated with a decreasing concentration of antisera before infection of cells. The amount of virus in the control wells was set to 100%. The 50% end-point titer (neutralizing dose [ND50]) virus-antiserum pair was determined and converted into mIU/mL. Values and error bars represent SD from two independent experiments (Used with permission of Mayo Foundation for Medical Education and Research).

A high neutralizing antibody response against the homotypic virus was observed when guinea pig antisera were used after MeV immunization (Figure 5A, virus #1). Yet, no cross-reactivity was noted against CDV-envelope pseudotyped rMeV (virus #6). Single swap of MeV-H by the counterpart CDV-H completely (virus #2) eliminated neutralization titers present in the antisera. Residual neutralization activity was observed only when PRMN was performed on Vero/NECTIN-4 cells. Only the absence of the MeV-H globular head (virus #3) sufficed to eliminate all neutralizing activity. As a consequence, neutralizing activity was observed when only the MeV-H globular head was present (virus #8). In the assumption of marginal reactivities to the MeV-H stalk and MeV-F, the joint presence would be expected to result in a marked difference in plaque reduction microneutralization (PRMN) titers. However, we did not see any, regardless of the entry receptor expressed on the cell (virus #3).

Similar to the case of the guinea pig antisera, ferrets immunized with CDV showed a highly protective neutralizing response for the homotypic virus (virus #6) (Figure 5B). Unlike the case of guinea pig antisera, substitution of CDV-H for the MeV-H homolog (virus #5) did not result in a decreased neutralization sensitivity of the virus. Of note, substitution of CDV-F (virus #2) caused a 4.5 and 3.3 log_2_ reduction in PRMN titers in a SLAMF1- and NECTIN-4–dependent manner, respectively. A difference greater than 2 log_2_ that equals two antigenic units is broadly considered to distinguish between two antigenic groups [59,60]. Therefore, these results show that in ferrets, most neutralizing antibodies are elicited against F protein. Within CDV-H protein, the presence of the globular head seemed sufficient to maintain PRMN titers (virus #3). Nevertheless, residual neutralization titers were observed when the CDV-H globular head was replaced and only the CDV-H stalk was present (virus #4). Since no cross-reactivity was observed in the expression of the counterpart’s envelope glycoproteins of MeV, our data showed the presence of CDV-H stalk-reactive antibodies. 

For the measles-immune human antisera, reactivity for CDV-H and CDV-F was observed only at high concentrations of antisera (Figure 5, virus #6) and was more apparent when PRMN assay was performed on Vero/NECTIN-4. Nevertheless, the 50% end-point titer (neutralizing dose, ND50) was below the considered protective levels (210 mIU/mL [24]). Consequently, PRMN titers higher than 210 mIU/mL for any isogenic chimera with single exchange of an envelope glycoprotein was considered indicative of immunogenicity. Single substitution of CDV-F by MeV-F in the isogenic MeV pseudotyped with CDV-envelope glycoproteins (virus #2) resulted in PRMN titers above the protective levels, thereby unveiling neutralizing antibodies against MeV-F protein. Again, in the context of the rMeV pseudotyped with the CDV-envelope glycoproteins, grafting the MeV-H globular head but not the stalk domain resulted in protective levels of neutralizing antibodies (virus #8 vs #7). In line with this, the reverse constructs in which parental MeV included CDV-H stalk (virus #4) resulted in less than 1 log_2_ difference in PRMN titers. Substitution of MeV-F by CDV-F in the context of MeV-H expressing virus (virus #5) resulted in a PRMN titer reduction of 32% and 34% (an antigenic unit reduction of 0.5) on a NECTIN-4– and SLAMF1-dependent manner, respectively. Taken together, our results indicate that MeV-F and MeV-H globular heads are the main inducers of neutralizing antibodies in humans. Conversely, the F protein appears to be immunodominant for CDV. 

## 4. Discussion

Morbillivirus infection and vaccination induce H- and F-specific neutralizing antibodies. By using hybrid H proteins based on morbillivirus with non–cross-reactive antibodies, we have shown that the H globular head is the main antigenic determinant of this protein in both MeV and CDV. Further heterotypic combination of both H and F envelope proteins conclusively demonstrated the otherwise-underestimated presence of F-specific neutralization. Importantly, either H globular head–specific and F-specific antibodies confer neutralizing titers above the 210 mIU/mL level for protection. 

Transfection of heterotypic glycoprotein pairs usually results in impaired functionality, but successful interchangeability has been achieved for closely related species such as MeV and CDV [20,61,62,63]. Virus strain seems critical for heterologous compatibility [64]. We have expanded these observations since neither wild-type/wild-type nor wild-type/vaccine combinations of MeV and CDV resulted in the production of fusion complementation (Muñoz-Alía and Russell, unpublished data, 2019), and only vaccine combinations thereof worked (i.e., this study). The strength of envelope protein interaction is known to affect fusion activity [65,66]. Yet, we did not pursue assessment of the H and F physical interaction since our efforts to detect equally the F glycoprotein of the two viral species by means of a FLAG-tag specifically abrogated heterotypic complementation (Muñoz-Alía and Russell, unpublished data, 2019) [67].

The stalk region of H protein has a key role in the activation signaling required to trigger F [64,68]. Since we observed heterotypic complementation between MeV and CDV glycoproteins, we expected hybrid H proteins to stay functional and to activate F triggering in a heterotypic manner. However, whereas the hybrid MeV/CDV-H induced fusion when coexpressed with MeV-F, it did not have this induction effect when CDV-F was used. Talekar et al. [69] reported that a hybrid H composed of the MeV-H stalk and Newcastle disease virus globular head promoted MeV-F–mediated fusion. In contrast, Singethan et al. [16] did not observe syncytium formation when either MeV or CDV-H proteins had heterologous ectodomains, even though similar cell surface expression was detected. The apparent inability of the hybrid MeV/CDV-H to activate CDV-F as it did with MeV-F might have been the consequence of an apparent higher activation energy barrier for CDV-F [70]. 

Avila et al. [68] recently reported that a decrease in the activation energy of F protein required for its refolding process could allow H and F exchangeability. Since different receptor interactions could potentially provide the extra energy required, we determined the heterotypic functionality in different cell lines expressing natural or engineered receptors. In using the latter system, we did not observe an increase in fusion activity when Vero cells expressing the anti-His antibody were used instead of the parental CD46+ Vero cells. We have showed that increasing the affinity of H for the engineered receptor did not prove otherwise. This result suggests that the binding affinity of the antibody to the 6 × His peptide is already beyond the threshold for fusion to occur [56]. However, expression of NECTIN-4 and SLAMF1, whose binding affinity for H does not exceed that of the anti-6 × His scFv/6 × His binding affinity, improves it [57,71]. Significantly higher levels of steady-state cell surface expression could be regarded as a potential explanation (both anti-His × 6 scFv and SLAMF1 carried identical HA tags), but other factors such as receptor protein length or outside-in effect cannot be ruled out [72,73]. 

Despite impairment in the fusion activity of the MeV/CDV-H CDV-F combination, as seen by cotransfection of DNA-expressing plasmids, virus chimeras expressing the counterpart envelope were recovered and shown to be functional. A possible explanation for the newly observed phenotype could be offered in the context of a looser interaction with the heterologous M proteins, which would enhance fusion activity [40,74]. Although H, F, and M interaction must be compatible at some extent between MeV and CDV, they are not optimal, as suggested by a lower virus rescue efficiency and growth rates of the chimeric viruses. Beaty et al. [48] recently reported a significant increase in the robustness and efficiency of the MeV rescue system, which allowed them to construct unbiased genome-wide mutagenesis libraries of recombinant viruses [75]. An improvement in the MeV reverse genetic system was shown herein to provide a clear advantage in the recovery of the chimeric viruses. As reported previously from the same group, switching one HHRbz for another one that was optimized for low Mg^2+^ concentration did not improve the rescue efficiency [52]. Differently, however, we obtained a significant increase when we combined the T7_opt_ promoter with an RNA pol II promoter. We have not compared whether both promoters acted additively, but the substitution of the T7 promoter for a human RNA polymerase II has resulted previously in an increase in the rescue efficiency [76]. 

While morbillivirus interacts efficiently with its species–specific receptor, interaction with homologous proteins of different species can be less efficient [10,11,58]. Hence, artificial inoculation has long been exploited in the morbillivirus vaccine to induce cross-protection [77,78,79]. Holzer et al. [78] found that cattle inoculated with wild-type peste des petits ruminants virus (PPRV) were protected from challenge with Rinderpest virus (RPV). Similarly, MeV vaccine has been used to protect puppies against CDV when acquired maternal antibodies interfere with CDV vaccination [80,81]. However, the cross-protection in the latter case has been more ascribed to a cell-mediated than humoral-mediated immunity [77,79,81]. Likewise, infection of macaques with either MeV or CDV failed to induce cross-neutralizing antibodies [4]. The H and mature F proteins of the MeV and CDV strains used in our study share 36% and 67% aa homology. Considerably more homology exists for the envelope glycoproteins of Hendra and Nipah virus: 83% and 88%, for which antisera does cross-neutralize [82]. 

The minimum homology threshold for a monotypic virus strain is estimated to be 80% [83]. Interestingly, viruses with as little as 33% homology in an envelope glycoprotein can still be cross-neutralized [84]. We observed low levels of CDV cross-neutralization only when using high-concentration MeV-immune antisera. This outcome was especially true when the neutralization assay was performed on Vero/NECTIN-4. This could help explain the lack of detection of cross-neutralizing antibodies in previous studies, although a lower titer of neutralizing antibodies against the homologous virus is an alternative explanation. In fact, Zhang et al. [7] observed a positive correlation between cross-CDV neutralization in human serum samples and anti-MeV antibodies neutralizing titers. Our consistently observed higher titers of neutralization with the use of Vero/NECTIN-4 cells vs Vero/SLAMF1 could be considered indicative of the preferential recognition of the SLAMF1 binding site for neutralizing antibodies.

The lack of cross-neutralization between MeV and CDV allowed us to decipher the envelope-specific neutralizing response that arose in animals and humans. Thus, we have shown in humans that both MeV-H and MeV-F induce protective levels of neutralizing antibodies, whereas MeV-H neutralizing antibodies were induced only by guinea pigs. By comparison, ferrets induced antibodies against CDV-F and CDV-H. Interestingly, anti–H stalk antibodies were also detected. 

In relation to the study findings, Ader-Ebert et al. [85] found a CDV-H stalk-directed monoclonal antibody that blocked syncytia formation. We thus have herein expanded their finding and extend it to MeV-H. Our study also corroborates our recent work with an antibody depletion approach [86]. There, selective depletion of MeV-H–specific antibodies with stably transfected cell lines did not result in loss of protective neutralization titers in human sera. As to the striking lack of anti–MeV-F antibodies in guinea pig, they definitely were present in HuCD46Ge-IFNar^KO^ mice (Muñoz-Alía and Russell, unpublished data), but they were a minor fraction of the total of neutralizing antibodies. This subdominance could well explain the scarcity of MeV-F–specific monoclonal antibodies in the literature. In humans, however, if we consider that neutralization titers conferred by the measles vaccine do not suffice for sterile immunity [87,88], a subclinical infection with a different measles genotype would skew the neutralizing antibody response toward MeV-F, which is much more conserved across genotypes. 

A clear limitation of the present study is the lack of comparable conditions in the immunization protocols that could influence the immunodominance [89]. Future studies will try to address this variability. Previous reports have shown H-specific antibodies as the main correlate of protection [90,91]. The H gene is one of the most genetically variable genes in the genome and, in consequence, is granted more relevance in antigenic drift studies. Because a 9.6% change in the aa sequence translates into an antigenically significant difference [59], the expectation is that CDV but not MeV genotypes will be differentiated in cross-neutralization assay with H antisera. Since mature F protein aa divergence is much lower than that for H, one can speculate that targeting the F protein would have a more important role in sustaining the morbillivirus monotypic nature. In support of this, da Fontoura Budaszewski et al. [31] recently reported their finding that immunization of ferrets with the vaccine CDV-H induced an antibody response against the vaccine but not against a wild-type strain. Consequently, the ferrets died during a lethal challenge. Instead, vaccination with both vaccine CDV-H and F protected ferrets from wild-type CDV [31]. 

Of the possible mechanisms of antibody-mediated neutralization, we focused only on direct neutralization of virus by antibodies in the present study. The striking difference on the presence of MeV and CDV-H-stalk neutralizing antibodies could have resulted from a different mechanism of action in the former. Further work on a role of antibody-dependent and complement-dependent cytotoxicity should clarify this hypothesis. 

MeV is thought to be the result of a spillover transmission into humans from the now-eradicated RPV [92]. Both recent CDV host-range expansion and identification of single aa changes in the H proteins of CDV and PPRV that allow them to use human receptors highlight the potential for new morbilliviruses to move into vacated ecological niches [3,6,58,93]. Because the current MeV vaccine does not protect against CDV infection, the development of new vaccines that bestow sterilizing immunity against other veterinary morbilliviruses is needed before MeV eradication can be achieved [4,5]. 

We believe that our rMeV encoding MeV-H and CDV-F will lay the groundwork for the development of such vaccines. The tools described herein provide the opportunity to identify other antigenic determinants in related morbilliviruses, for the ultimate development of pan-protective vaccines against another morbilliviruses. Such a concept has recently been exploited in the newly created *Pneumoviridae* family, separate from the original *Paramyxoviridae* family [94,95]. Thus, chimeric fusion protein consisting of respiratory syncytial virus and human metapneumovirus were shown in mice to induce cross-neutralizing immune responses [94,95].

In summary, we have described functional chimerism between MeV and CDV envelope glycoproteins. With this tool, we were able to show that vaccination or natural infection could induce potent H- and F-reactive neutralizing antibodies, or both. H hybrids further unveiled the presence of H-stalk neutralizing antibodies. Additional chimerism among morbilliviruses could be used not only to unveil antigenic determinants involved in protection but also to ultimately create multivalent vaccines against morbillivirus with zoonotic potential.

## 5. Conclusions

Morbilliviruses induce H- and F-specific neutralizing antibodies. The globular head is the main antigenic determinant of the H protein. Either H- or F-specific antibodies can induce protective titers of neutralizing antibodies, but immunodominance is species specific or host related, or both.

Chimeric morbilliviruses can be used to quantitatively and qualitatively interrogate the cross-reactome on vaccination or natural infection, or both. Virus chimera can ultimately be used as multivalent vaccine against morbillivirus with zoonotic potential.

## Abbreviations

aa	amino acid
ATCC	American Type Culture Collection
BHK	baby hamster kidney
CDV	canine distemper virus
CDV-F	canine distemper virus with F glycoprotein
CDV-H	canine distemper virus with H glycoprotein
DSP	dual-split reporter plasmid
EGFP	enhanced green fluorescent protein
FACS	fluorescent activated cell sorting
FBS	fetal bovine serum
FFU	fluorescence-forming unit
GFP	green fluorescent protein
HA	hemagglutinin
HDVRbz	hepatitis delta virus ribozyme
HHRbz	hammerhead ribozyme
HRP	horseradish peroxidase
Ig	immunoglobulin
MeV	measles virus
MeV-F	measles virus with F glycoprotein
MeV-H	measles virus with H glycoprotein
NECTIN-4	nectin cell adhesion molecule 4
NGS	N-linked glycosylation sites
PCR	polymerase chain reaction
PPRV	peste des petits ruminants virus
PRMN	plaque reduction microneutralization
PVDF	polyvinylidene fluoride
rMeV	recombinant measles virus
RPMI	Roswell Park Memorial Institute
RPV	Rinderpest virus
scFv	single-chain variable fragment
SLAMF1	signaling lymphocytic activation molecule family member 1
T7t	T7 terminator
